# Expression of Eph receptor A10 is correlated with lymph node metastasis and stage progression in breast cancer patients

**DOI:** 10.1002/cam4.156

**Published:** 2013-11-07

**Authors:** Kazuya Nagano, So-ichiro Kanasaki, Takuya Yamashita, Yuka Maeda, Masaki Inoue, Kazuma Higashisaka, Yasuo Yoshioka, Yasuhiro Abe, Yohei Mukai, Haruhiko Kamada, Yasuo Tsutsumi, Shin-ichi Tsunoda

**Affiliations:** 1 Laboratory of Biopharmaceutical Research, National Institute of Biomedical Innovation7-6-8 Saito-Asagi, Ibaraki, Osaka, 567-0085, Japan; 2 Graduate School of Pharmaceutical Sciences, Osaka University1-6 Yamadaoka, Suita, Osaka, 565-0871, Japan; 3 The Center for Advanced Medical Engineering and informatics, Osaka University1-6 Yamadaoka, Suita, Osaka, 565-0871, Japan

**Keywords:** Breast cancer, Eph receptor A10, lymph node metastasis

## Abstract

Eph receptor A10 (EphA10) is a valuable breast cancer marker that is highly expressed in breast cancer tissues by comparison with normal breast tissues, as we previously reported. However, the role of EphA10 expression in breast cancer is not well understood. Here, we have analyzed the expression of EphA10 at the mRNA- and protein-level in clinical breast cancer tissues and then evaluated the relationship with clinicopathological parameters for each sample. EphA10 mRNA expression was quantified by real-time polymerase chain reaction using complimentary DNA (cDNA) samples derived from breast cancer patients. Lymph node (LN) metastasis and stage progression were significantly correlated with EphA10 expression at the mRNA level (*P* = 0.0091 and *P* = 0.034, respectively). Furthermore, immunohistochemistry (IHC) staining of breast cancer tissue microarrays (TMAs) revealed that EphA10 expression at the protein level was also associated with LN metastasis and stage progression (*P* = 0.016 and *P* = 0.011, respectively). These results indicate that EphA10 expression might play a role in tumor progression and metastasis. Our findings will help elucidate the role of EphA10 in clinical breast cancer progression.

## Introduction

Eph receptors comprise the largest subgroup of the receptor tyrosine kinase family of proteins. Currently, nine type-A (EphA1–A8, EphA10) and five type-B (EphB1–B4, EphB6) molecules are known in mammals. Eph family receptors play important roles in physiological development such as neural development [1] and glucose homeostasis [2]. In addition, several Eph family receptors were implicated in various aspects of the tumor malignancy, including tumorigenesis [3, 4], proliferation [5, 6], vasculogenesis [7, 8] or metastasis [9–11]. Indeed, EphA2 is highly expressed in several kinds of tumor, and this enhanced expression is thought to be related to tumor progression [3, 5, 9, 10]. Currently, clinical trials of a EphA2-targeting drug are ongoing [12]. Therefore, the expression profiles, function, and targeting therapy for Eph family receptors are directly related to cancer biology and drug development.

EphA10 is a novel breast cancer marker that was originally discovered by ourselves using a proteomics approach [13]. Prior to this discovery, EphA10 was only known to be expressed in the testis at the mRNA level [14]. Our group has developed an “antibody proteomics system”, which facilitates the validation of biomarker candidates identified from proteome analyses [13]. Using this method, we previously revealed that EphA10 is expressed in many breast cancer tissues compared to normal tissues [13]. However, the function of EphA10 has not been fully analyzed. Consequently, the relationship between EphA10 and clinical tumor progression is poorly understood.

Here, we first analyzed the statistical relationship between EphA10 mRNA expression in clinical tumor tissues and their clinicopathological parameters. Next, we evaluated the correlation with EphA10 expression at the protein level, which is important to fulfill the EphA10 function. This data will help elucidate the role of EphA10 in clinical breast cancer progression.

## Material and Methods

### Analysis of EphA10 mRNA expression by real-time polymerase chain reaction

**Figure 1:**
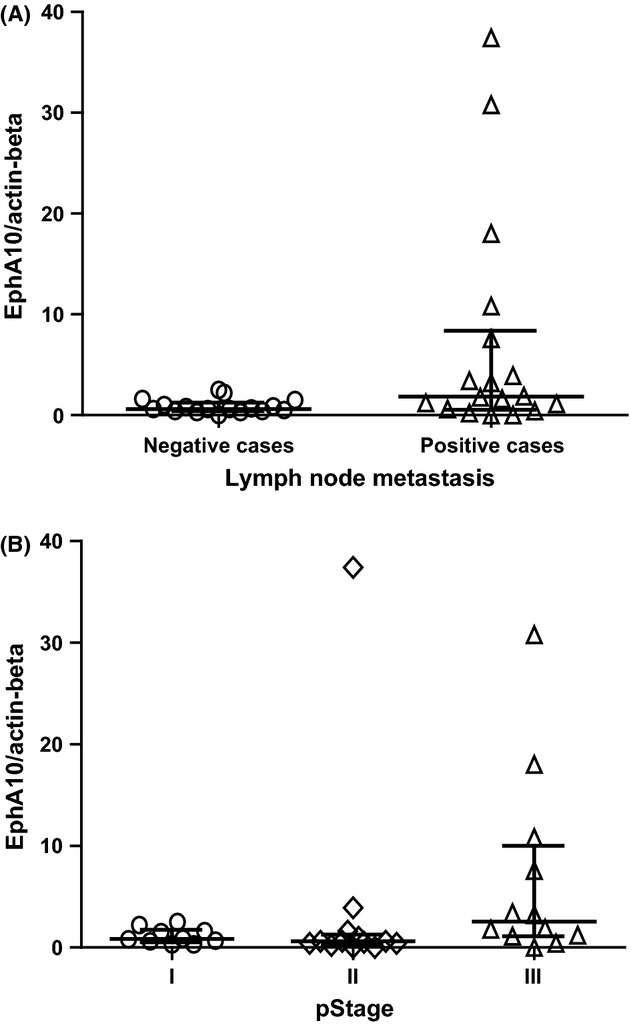
EphA10 mRNA expression level analysis in lymph node (LN)-positive and -negative cases, or stage I, II, and III. EphA10 mRNA expression level in each case was normalized to that of actin-beta. The ratio of EphA10 mRNA expression level against median value was plotted for LN-positive and -negative cases (A), or stage I, II, and III, respectively (B). Differences were evaluated using the Mann–Whitney test (*P* = 0.025) (A) and Kruskal–Wallis test (*P* = 0.044) (B). Bar and range show the median with interquartile range in each group.

Complimentary DNAs (cDNA) derived from human breast tumors were purchased from OriGene Technologies (Rockville, MD). The 20 *μ*L polymerase chain reaction (PCR) mixture included 1 *μ*L of cDNA template, 10 *μ*L of TaqMan Gene Expression Master Mix, and 1 *μ*L of TaqMan probe (EphA10:Hs01017018_m1 or actin-beta:Hs99999903 _m1) (Life Technologies, Carlsbad, CA). Reactions were performed according to the manufacturer's instructions. The threshold cycles were determined using the default settings. EphA10 mRNA expression levels were normalized against actin-beta. Cases with greater or less than the median value were classified into a high or low expression group, respectively (Table 1). In Figure 1, we display the EphA10 mRNA expression level as the ratio against the median.

**Table 1:** Correlation between EphA10 mRNA expression and clinicopathological characteristics.

Characteristics	*n*	EphA10 mRNA expression		*P* value	
	
		High *n* (%)	Low *n* (%)	*χ*^2^	Mann–Whitney
Age					
<45	5	2 (40.0)	3 (60.0)	1.00	–
≧45	30	15 (50.0)	15 (50.0)		
Gender					
Male	0	0 (0.0)	0 (0.0)	–	–
Female	35	17 (48.6)	18 (51.4)		
Histological classification					
Invasive ductal carcinoma	32	15 (46.9)	17 (53.1)	0.21	–
Invasive lobular carcinoma	2	2 (100.0)	0 (0.0)		
Squamous cell carcinoma	1	0 (0.0)	1 (100.0)		
pT					
T1	13	6 (46.2)	7 (53.8)	0.23	0.25
T2	18	8 (44.4)	10 (55.6)		
T3	3	3 (100.0)	0 (0.0)		
T4	1	1 (100.0)	0 (0.0)		
pN					
N0	17	5 (29.4)	12 (70.6)	0.045	0.0091
N1	9	6 (66.7)	3 (33.3)		
N2	5	3 (60.0)	2 (40.0)		
N3	4	4 (100.0)	0 (0.0)		
pStage					
I	10	4 (40.0)	6 (60.0)	0.022	0.034
II	13	4 (30.8)	9 (69.2)		
III	12	10 (83.3)	2 (16.7)		

Indication of each pathological parameter is as follows: pT, degree of size of the primary tumor; pN, degree of spread to regional lymph nodes; pStage, degree of cancer progression.

### Analysis of EphA10 protein expression by immunohistochemistry staining

Breast cancer tissue microarrays (TMAs) (US Biomax, Rockville, MD) were deparaffinated in xylene and rehydrated in ethanol. Epitope retrieval was performed by maintaining the Target Retrieval Solution (Dako, Glostup, Denmark) according to the manufacturer's instructions. After treatment, endogenous peroxidase was blocked with 0.3% H_2_O_2_ for 5 min. The slides were then incubated with anti-human EphA10 polyclonal antibody (Abgent, San Diego, CA) for 30 min. After washing three times, the slides were incubated for 30 min with Envision+Dual Link (Dako, Glostup, Denmark). Finally, the slides were washed three times and treated in 3,3′-diaminobenzidine and counterstained with hematoxylin. For statistical analysis, study samples were divided into high and low expression groups based on the following two criteria. In terms of distribution, the percentage of positive cells in all tumor cells was scored as 0 (0%), 1 (1–50%), and 2 (51–100%). In terms of quantity, the signal intensity was scored as 0 (no signal), 1 (weak), 2 (moderate), or 3 (marked). Cases with a total score of ≥3 were classified into the high expression group.

### Statistical methods

All analyses were performed using GraphPad Prism 5 version (GraphPad Software Inc., La Jolla, CA). Chi-square or Fisher's exact test were used to compare the categorical variables. Differences between two or three groups were analyzed by the Mann–Whitney or Kruskal–Wallis test, respectively. All hypothesis testing was two-tailed with a significant level of 0.05.

## Results and Discussion

In order to analyze the contribution of EphA10 to clinical breast cancer progression, we evaluated a possible correlation between EphA10 mRNA expression in the clinical tumor tissues and clinicopathological parameters such as primary tumor size (pT), lymph node (LN) metastasis (pN) and stage grouping as indicators of cancer progression (Table 1). Statistical analysis showed that EphA10 expression was independent of age, histological classification, and pT indexes. Nonetheless, EphA10 mRNA expression was positively associated with the progression of the stage, which strongly supports our previous TMA-based analysis [13]. Furthermore, we found that EphA10 expression was also positively correlated with LN metastasis. Given that a combination of pT and pN values in each case defines stage I–III, these data suggest a significant correlation with LN metastasis might contribute to that with stage progression.

For detailed evaluation of the contribution of EphA10 expression to LN metastasis, we divided all the cases into LN metastasis positive and negative, and then plotted the EphA10 expression levels for the two groups. Figure 1A indicates that patients with elevated levels of EphA10 expression in tumor tissues were positive for LN metastasis. Indeed, a significant difference between LN-positive and -negative cases was observed. Moreover, we similarly analyzed for pStage. Figure 1B indicated that, with the exception of one outlier observed in stage II, EphA10 expression also displayed a significant positive correlation with stage progression. Taken together, these data suggest that the level of EphA10 expression partly contributes to breast cancer progression.

**Figure 2:**
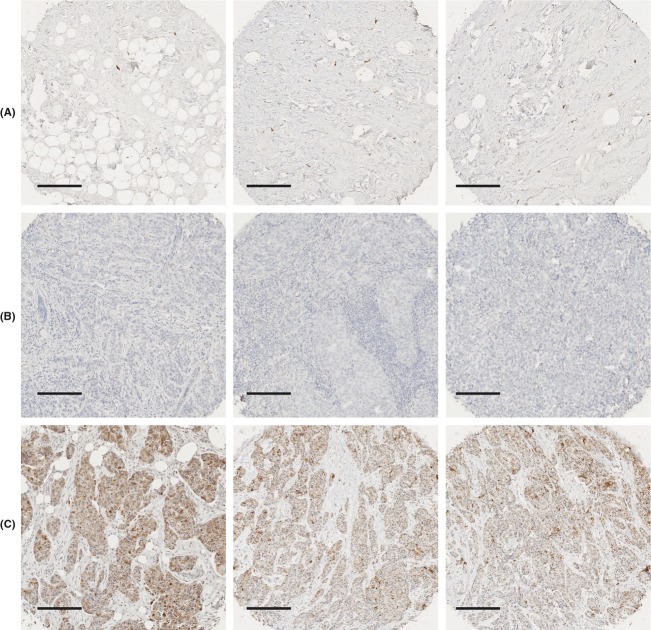
Immunohistochemical staining images in tissue microarray (TMA) with breast tumor and normal tissues. TMAs with breast tumor and normal tissues were stained using anti-EphA10 antibody. Representative images of normal breast tissue (A), EphA10 negative breast cancer tissue (B), and EphA10 positive breast cancer tissues (C) are shown. Scale bar: 200 *μ*m.

Next, we evaluated the relationship between EphA10 protein expression and clinicopathological characteristics. Immunohistochemistry (IHC) staining of TMAs showed that EphA10 was expressed in approximately 60% of breast tumor tissues, but not in normal breast tissues, which is consistent with our previous studies [13] (Fig. 2).

Statistical analysis revealed that EphA10 expression at the protein level was also independent of age, gender, histological classification and pT indexes. However, EphA10 protein expression was positively associated LN metastasis and stage progression (Table 2). Moreover, in order to validate this correlation, we also performed an analysis using a different anti-EphA10 antibody that we had isolated from a naïve phage antibody library, which gave similar results (data not shown). In addition, we also analyzed the possible correlation between EphA10 protein level and LN metastasis or stage progression, using the IHC staining total score as an indicator of the protein expression level. As shown to Figure S1, the total score is significantly associated with LN metastasis, but not stage progression. Taken together, our findings indicate that EphA10 expression is related to LN metastasis as well as stage progression in breast cancer, although improved quantitative analysis of protein expression level by mass spectrometry is needed.

**Table 2:** Correlation between EphA10 protein expression and clinicopathological characteristics.

Characteristics	*n*	EphA10 protein expression		*P* value	
	
		High *n* (%)	Low *n* (%)	*χ*^2^	Mann–Whitney
Age					
<45	103	61 (59.2)	42 (40.8)	0.19	–
≧45	199	133 (66.8)	66 (33.2)		
Gender					
Male	2	2 (100.0)	0 (0.0)	0.54	–
Female	300	192 (64.0)	108 (36.0)		
Histological classification					
Invasive ductal carcinoma	272	177 (65.1)	95 (34.9)	0.59	–
Invasive lobular carcinoma	10	5 (50.0)	5 (50.0)		
Invasive papillary carcinoma	6	5 (83.3)	1 (16.7)		
Mucinous carcinoma	2	1 (50.0)	1 (50.0)		
Medullary carcinoma	2	2 (100.0)	0 (0.0)		
Carcinosarcoma	1	1 (100.0)	0 (0.0)		
pT					
T1	21	15 (71.4)	6 (28.6)	0.35	0.96
T2	200	127 (63.5)	73 (36.5)		
T3	46	26 (56.5)	20 (43.5)		
T4	35	26 (74.3)	9 (25.7)		
pN					
N0	154	90 (58.4)	64 (41.6)	0.044	0.016
N1	116	80 (69.0)	36 (31.0)		
N2	26	22 (84.6)	4 (15.4)		
N3	6	3 (50.0)	3 (50.0)		
pStage					
I	9	4 (44.4)	5 (55.6)	0.037	0.011
II	232	143 (61.6)	89 (38.4)		
III	61	47 (77.0)	14 (23.0)		

Indication of each pathological parameter is as follows: pT, degree of size of the primary tumor; pN, degree of spread to regional lymph nodes; pStage, degree of cancer progression.

Other Eph receptors such as EphA2 or EphB3 have invasive, migrating and anoikis-inhibiting abilities, so that EphA2- or EphB3-expressing cancer cells promote metastasis [3, 9–11]. Thus, EphA10 could also elicit a similar biological effect on disease progression, although further investigations are needed to elucidate the mechanism by which EphA10 expression is correlated with LN metastasis. Moreover, aiming for LN metastasis prediction, we should set up the criteria by more sample analyses and evaluate LN metastasis prospectively.

In conclusion, we demonstrated that EphA10 expression at both the gene and protein level in clinical breast cancer tissues is significantly associated with LN metastasis as well as stage progression. We believe that the data will help elucidate the biological function of EphA10 and facilitate the development of novel breast cancer drugs.

## Supporting Information

**Figure S1.** EphA10 protein expression level analysis in LN-positive and -negative cases, or stage I, II, and III.
